# Kratom-Associated Pulmonary Toxicity: A Systematic Review of Case Reports and Forensic Series with a Proposed Clinical Phenotype Classification

**DOI:** 10.1007/s13181-026-01153-5

**Published:** 2026-08-27

**Authors:** Venkatkiran Kanchustambham, Swetha Saladi

**Affiliations:** 1 Pulmonary & Critical Care Medicine, Sanford Health, Fargo, North Dakota 58122 USA; 2 University of North Dakota School of Medicine & Health Sciences, Fargo, North Dakota USA

**Keywords:** Kratom, Mitragynine, 7-hydroxymitragynine, Pulmonary toxicity, Diffuse alveolar hemorrhage

## Abstract

**Introduction:**

Kratom use has increased, including concentrated 7-hydroxymitragynine products, but pulmonary toxicity has not been systematically synthesized.

**Methods:**

We systematically reviewed human case reports, conference abstracts, and forensic/autopsy series describing pulmonary outcomes after kratom, mitragynine, or 7-hydroxymitragynine exposure (PROSPERO CRD420261427143). Five databases were searched through 24 June 2026. Two reviewers independently screened, extracted, and appraised reports; causality was graded using Naranjo criteria for clinical reports and WHO-UMC criteria for forensic reports. Synthesis was narrative.

**Results:**

Twenty-four reports were included: 14 clinical and 10 forensic. Five categories were identified in two tiers: primary pulmonary syndromes—diffuse alveolar hemorrhage (*n* = 3), acute respiratory distress syndrome (ARDS)/diffuse acute lung injury (*n* = 3), and eosinophilic/hypersensitivity pneumonitis (*n* = 2)—and secondary consequences of systemic toxicity—central respiratory depression (*n* = 6) and post-mortem pulmonary edema/congestion (*n* = 10). All clinical patients survived; all forensic decedents died. Attribution was Probable in 9 reports, Possible in 13, and Unlikely in 2. Pulmonary edema/congestion was the most common forensic finding but was nonspecific; single-agent fatalities had high mitragynine concentrations (2,325–7,500 ng/mL).

**Conclusions:**

Reported kratom-associated pulmonary presentations cluster into five provisional, hypothesis-generating categories spanning primary lung injury and secondary systemic effects. Clinicians should consider kratom and 7-hydroxymitragynine in unexplained acute lung injury, alveolar hemorrhage, eosinophilic pneumonitis, or opioid-type respiratory depression; targeted toxicology and standardized reporting are needed to refine this framework.

**Registration:**

PROSPERO CRD420261427143.

## Introduction

Kratom (Mitragyna speciosa) is a tropical tree of Southeast Asia whose leaves contain the psychoactive indole alkaloids mitragynine and the substantially more potent 7-hydroxymitragynine. These alkaloids act as partial mu-opioid receptor agonists with additional adrenergic and serotonergic activity, producing stimulant effects at low doses and opioid-like effects at higher doses [1, 2]. 7-hydroxymitragynine constitutes only a small fraction of the alkaloid content of the native leaf — approximately 1–2% of total leaf alkaloids [2] — yet is a far more potent mu-opioid agonist than mitragynine; concentrated and semi-synthetic 7-hydroxymitragynine products therefore differ fundamentally from traditional leaf preparations in expected toxicity [2]. In the United States kratom is increasingly used to self-treat pain, anxiety, and opioid withdrawal, and is widely available as leaf, powder, capsule, brewed tea, extract, and, more recently, concentrated semi-synthetic 7-hydroxymitragynine products [3, 4].

Reports of kratom-related toxicity to poison-control centers have risen steeply over the past decade [3], and in 2025 the U.S. Food and Drug Administration recommended scheduling 7-hydroxymitragynine [4], reflecting growing concern about high-potency products. Kratom-associated hepatic, cardiovascular, and neurological injury has been reviewed at the organ-system level [2, 5], but pulmonary injury has appeared only as isolated case reports, conference abstracts, and incidental autopsy findings, and has never been systematically synthesized.

This gap is clinically important. The differential diagnosis of acute lung injury is broad, kratom use is easily missed unless specifically elicited, and forensic series report pulmonary edema and congestion as a recurring — though nonspecific — finding in fatal cases [5]. We therefore systematically identified and synthesized published reports of pulmonary manifestations associated with kratom, characterized their clinical, radiological, bronchoscopic, and pathological features, proposed a provisional phenotype classification, and appraised the strength of evidence for causation while accounting for polysubstance exposure.

## Methods

### Protocol and Registration

The review was registered prospectively (PROSPERO CRD420261427143) and is reported in accordance with the PRISMA 2020 statement [6]. A narrative synthesis was planned a priori; no meta-analysis was performed, reflecting the case-level, heterogeneous nature of the evidence. Although conducted and reported as a systematic review, the available evidence — case reports, conference abstracts, and retrospective forensic series — is characteristic of that encountered in a scoping review, and our synthesis is correspondingly framed as descriptive and hypothesis-generating.

### Eligibility Criteria

We included case reports, case series, published conference abstracts, and forensic/autopsy series reporting at least one respiratory or pulmonary clinical, radiological, bronchoscopic, or pathological outcome in humans of any age exposed to kratom or to mitragynine/7-hydroxymitragynine, documented either clinically (history or confirmed product use) or analytically (alkaloid detection in blood, urine, or post-mortem specimens). No language or date restriction was applied. We excluded reports without a pulmonary outcome, non-human or in-vitro/in-silico studies, reports without documented kratom exposure, and narrative or systematic reviews without primary case data (used only for reference cross-checking and citation searching).

### Search and Screening

PubMed/MEDLINE, Embase, Scopus, Web of Science, and Google Scholar were searched from inception to 24 June 2026, combining botanical and chemical terms (kratom, Mitragyna speciosa, mitragynine, 7-hydroxymitragynine) with respiratory terms (lung, pulmonary, respiratory, acute respiratory distress syndrome (ARDS), alveolar hemorrhage, pneumonitis, pneumonia, pulmonary edema, respiratory failure, respiratory depression), supplemented by hand-searching reference lists and proceedings. Records were de-duplicated and screened in two phases (title/abstract, then full text) by two independent reviewers, with disagreements resolved by consensus. Exclusion reasons were applied hierarchically and the single highest-ranking reason recorded per record.

### Data Extraction, Phenotype Classification, and Appraisal

Two reviewers independently extracted data and cross-checked all values against the primary source. The primary outcome was the reported pulmonary presentation, classified into one or more of five pre-specified categories: (1) diffuse alveolar hemorrhage; (2) ARDS or other diffuse acute lung injury; (3) eosinophilic or hypersensitivity-type pneumonitis; (4) opioid-like central respiratory depression; and (5) pulmonary edema or congestion. For interpretation, we group categories 1–3 as primary pulmonary syndromes, in which the lung is the apparent target organ, and categories 4–5 as secondary pulmonary consequences of systemic toxicity, in which the lung is the effector organ (category 4) or the site of a nonspecific terminal finding (category 5). Methodological quality was appraised with the Murad et al. (2018) tool for case reports and series [7]. Because attribution is complicated by frequent polysubstance exposure, the exposure–outcome relationship was additionally graded for each clinical report with the Naranjo scale [8] and for each forensic case with the WHO-UMC system [9]; the limitations of both instruments in this setting are considered in the Discussion. The lead reviewer is a co-author of one included report; that report was screened, appraised, and causality-graded independently by the second reviewer, and the lead reviewer was recused from its assessment. The two reviewers rated independently and reconciled discrepancies by consensus; a formal inter-rater agreement statistic (e.g., Cohen’s kappa) was not calculated, which we note as a limitation.

### Synthesis

Findings were summarized descriptively by category, with categorical variables as counts and proportions and continuous variables as medians and ranges. Pre-specified groupings compared clinical versus forensic setting and single-substance versus polysubstance exposure. As qualitative robustness checks, conclusions were re-examined after restricting to reports graded Probable or better, to single-substance exposures, and to peer-reviewed clinical reports (excluding conference abstracts).

## Results

### Study Selection

**Fig. 1:**
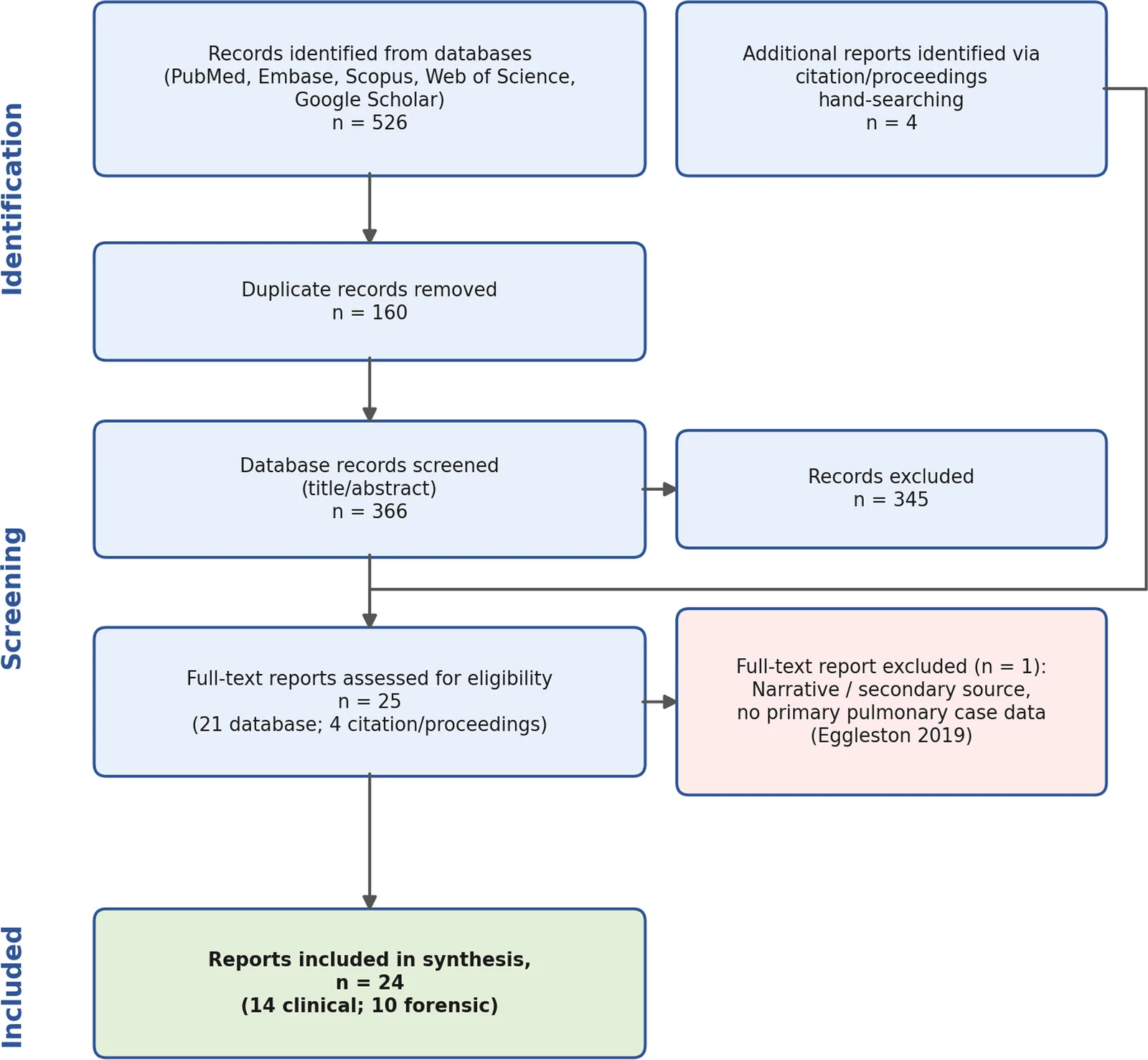
PRISMA 2020 flow diagram for the systematic review of kratom-associated pulmonary toxicity (PROSPERO CRD420261427143). Of 526 records identified from databases and 4 from citation/proceedings hand-searching, 24 reports were included in the synthesis (14 clinical, 10 forensic)

The search yielded 526 database records. After removal of 160 duplicates, 366 database records were screened by title and abstract, and 345 were excluded. Twenty-one database reports proceeded to full-text assessment. Four additional reports identified through citation/proceedings hand-searching were also assessed, resulting in 25 full-text reports assessed for eligibility. One report was excluded as a secondary/aggregate source without extractable individual pulmonary case data, leaving 24 reports for synthesis (Fig. 1).

### Study Characteristics

Twenty-four reports were included: 14 clinical (9 full case reports, 5 conference abstracts), each describing a single patient, and 10 forensic reports (single cases and autopsy series). Eight forensic reports describe one or two decedents each (Domingo et al. [10] report two fatalities, of which one had a pulmonary finding); two are larger autopsy series — Mata & Andera [11] (20 decedents) and Jittasopa & Srisont [12] (24 mitragynine-positive decedents, 7 with pulmonary edema and 4 with severe pulmonary congestion). The unit of synthesis is the report, and phenotype counts (Tables 1 and 2) refer to reports rather than to individual decedents. Reports spanned 2011–2026 and were predominantly from the United States. Among single-patient reports the median age was 32 years (range 17–69), and 68% were male. Case-level characteristics are summarized in Table 1 [10–33].

**Table 1:** Characteristics of the 24 included reports. CR, case report; Abs, conference abstract; DAH, diffuse alveolar hemorrhage; ARDS, acute respiratory distress syndrome; UDS, urine drug screen; COD, cause of death; 7-OH, 7-hydroxymitragynine; EF, ejection fraction. Mitragynine concentrations are post-mortem blood unless stated. c1, case 1: Domingo et al. reported two fatalities, of which only case 1 had a documented pulmonary finding and is included here

Study (year)	Country	Age / Sex	Type	Phenotype	Mitragynine / co-exposure	Outcome	Attribution grade (Naranjo—clinical; WHO-UMC—forensic)
Kanchustambham 2025	USA	mid / F	CR	DAH	Not measured; no co-exposure (workup neg; vaping excluded)	Survived	Probable
Weerakoon 2016	USA	22 / M	Abs	DAH	Not measured; benzodiazepines + opioids on UDS	Survived	Possible
Bharati 2025	USA	27 / M	CR	DAH	Not measured; LEPTOSPIROSIS (PCR+) = alternative cause	Survived	Unlikely
Minich 2021	USA	69 / F	Abs	Pneumonitis	Not measured; kratom only	Survived	Probable
Pathak 2014	USA	22 / M	Abs	ARDS	Not quantified; alcohol	Survived	Possible
Jaliawala 2018	USA	32 / F	Abs	ARDS	Not quantified; recent CAP	Survived	Possible
Chinnappan 2023	USA	36 / M	CR	ARDS	Not measured; no active co-exposure	Survived	Probable
Parekh 2023	USA	58 / F	Abs	Pneumonitis	Not measured; kratom only; BAL eosinophilia + positive rechallenge	Survived	Probable
Ahmed 2023	USA	40s / F	CR	Resp depression	Not measured; UDS negative (clean)	Survived	Probable
Duncan 2026	USA	38 / M	CR	Resp depression	Not measured; tianeptine + benzodiazepines	Survived	Possible
Pullman 2026	USA	29 / M	CR	Resp depression	7-OH product 190 mg ingested; alcohol + amphetamines	Survived	Possible
Overbeek 2019	USA	38 / F	CR	Resp depression	GC/MS kratom-only (no other opioids)	Survived	Probable
Palasamudram 2019	USA	36 / M	CR	Resp depression	Urine 7-OH > 500 ng/mL; tox otherwise neg	Survived	Probable
Bahji 2023	Canada	38 / M	CR	Resp depression	Not measured; benzodiazepine + cannabis; EF 35–40%	Survived	Possible
Behonick 2022	USA	37,33 / M	Forensic	Edema/congestion	2,325 & 3,809 ng/mL; SINGLE-DRUG (350 + screen neg)	Fatal	Probable
Mata 2023	USA	32 / M	Forensic	Edema/congestion	Central 7,500 ng/mL; only mitragynine; cardiomegaly/LVH	Fatal	Probable
Mata & Andera 2020	USA	mean 33	Forensic series	Edema/congestion	10 − 4,310 ng/mL (*n* = 20); all polysubstance (3 sole-COD)	Fatal	Possible
Wang 2018	Canada	56 / F	Forensic	Edema/congestion	2,500 ng/mL; oxycodone + lorazepam; COPD	Fatal	Possible
McIntyre 2015	USA	24 / M	Forensic	Edema/congestion	190/230 ng/mL; venlafaxine, mirtazapine, others	Fatal	Possible
Jittasopa 2021	Thailand	series	Forensic series	Edema/congestion	Quantified; variable co-exposures	Fatal	Possible
Holler 2011	USA	20 / M	Forensic	Edema/congestion	390 ng/mL; propylhexedrine = certified COD	Fatal	Possible
Neerman 2013	USA	17 / M	Forensic	Edema/congestion	600 ng/mL; DXM, diphenhydramine, temazepam	Fatal	Possible
Karinen 2014	Norway	17 / M	Forensic	Edema/congestion	1,060 ng/mL; zopiclone, citalopram	Fatal	Possible
Domingo 2017 (c1)	Germany	22 / M	Forensic	Edema/congestion	790 ng/mL; etizolam (toxic) + multiple psychotropics	Fatal	Unlikely

**Table 2:** Two-tier phenotype framework: characteristic features and high-attribution exemplars. Categories 1–3 are primary pulmonary syndromes in which the lung is the apparent target organ; categories 4–5 are secondary pulmonary consequences of systemic toxicity, in which the lung is the effector organ (category 4) or the site of a nonspecific terminal finding (category 5). BAL, bronchoalveolar lavage; ARDS, acute respiratory distress syndrome

Phenotype	*n*	Setting / outcome	Characteristic features	High-attribution exemplars (Probable+)
Tier A — Primary pulmonary syndromes (lung is the apparent target organ)				
1. Diffuse alveolar hemorrhage	3	Clinical; all survived	Hypoxemic failure, hemoptysis, bilateral ground-glass opacities, progressively bloody BAL	Kanchustambham (index)
2. ARDS / diffuse acute lung injury	3	Clinical; all survived	Bilateral infiltrates, low PaO2:FiO2, steroid- and prone-responsive; infection excluded; aspiration pneumonitis a potential mimic	Chinnappan
3. Eosinophilic / hypersensitivity pneumonitis	2	Clinical; all survived	BAL eosinophilia, subacute course, corticosteroid-responsive; positive rechallenge (Parekh); both reports are conference abstracts	Minich, Parekh
Tier B — Secondary pulmonary consequences of systemic toxicity (lung is the effector organ or the site of a nonspecific terminal finding)				
4. Central (opioid-like) respiratory depression	6	Clinical; all survived	Hypoventilation/hypercapnia, depressed consciousness, naloxone-responsive; imaging often normal; lung is the effector organ	Ahmed, Overbeek, Palasamudram
5. Pulmonary edema / congestion	10	Forensic; all fatal	Autopsy pulmonary edema/congestion, increased lung weight; a nonspecific terminal finding, common in opioid-type deaths generally	Behonick, Mata 2023 (single-drug)

### Pulmonary Phenotypes

Reports distributed across all five pre-specified categories, grouped here into two tiers (Table 2). Primary pulmonary syndromes — in which the lung appears to be the target organ — comprised diffuse alveolar hemorrhage (*n* = 3), ARDS/diffuse acute lung injury (*n* = 3), and eosinophilic/hypersensitivity pneumonitis (*n* = 2), and occurred exclusively in clinical reports. Secondary pulmonary consequences of systemic toxicity comprised central respiratory depression (*n* = 6) — a brainstem-mediated mu-opioid effect in which the lung is the effector organ, seen only in clinical reports — and pulmonary edema/congestion (*n* = 10), a nonspecific terminal finding confined to forensic cases. 

#### Diffuse Alveolar Hemorrhage (Primary)

Three clinical cases [13–15] presented with hypoxemic respiratory failure, bilateral ground-glass opacities, and progressively bloody bronchoalveolar-lavage aliquots; in the index case [13] an extensive autoimmune, infectious, and vasculitic workup was negative and vaping was excluded, whereas one case was ultimately attributed to leptospirosis (urine PCR-positive) and graded Unlikely [15]. 

####  ARDS / Diffuse Acute Lung Injury (Primary)

Three clinical cases [17–19] met ARDS criteria with bilateral infiltrates, low PaO2:FiO2, and exclusion of infection on bronchoalveolar lavage; all improved with lung-protective ventilation and corticosteroids, two after prone positioning. Aspiration pneumonitis, a common complication of depressed consciousness, can produce a similar radiographic picture and could not be excluded in every report. 

#### Eosinophilic / Hypersensitivity Pneumonitis (Primary)

Two cases [16, 20] showed subacute, corticosteroid-responsive pneumonitis; one demonstrated bronchoalveolar eosinophilia with a positive rechallenge on kratom re-exposure [20], the strongest single causal signal in the dataset. Both reports were conference abstracts, so this category rests entirely on non-peer-reviewed sources (see Sensitivity analyses). 

#### Central Respiratory Depression (Secondary)

Six clinical cases [21–26] presented with depressed consciousness, hypoventilation, and hypoxemia or hypercapnia, frequently naloxone-responsive; chest imaging was normal or non-specific in several, and one was accompanied by a non-ischemic cardiomyopathy that confounded attribution [26]. In this category the lung is the effector organ rather than the primary site of injury. 

#### Pulmonary Edema / Congestion (Secondary)

Ten forensic reports [10–12, 27–33] described pulmonary edema and/or congestion with increased lung weight at autopsy. This is a nonspecific terminal finding: pulmonary edema is present in the large majority of opioid-related deaths on post-mortem imaging [34], increased lung weight is not specific to opioids or to any single drug class [35], and in the largest kratom autopsy dataset pulmonary edema was recorded in roughly half of mitragynine-induced fatalities [36]. In the two single-drug fatalities [27], in which a 350-plus-analyte screen was otherwise negative, mitragynine concentrations were high (2,325 and 3,809 ng/mL; and 7,500 ng/mL central blood in a third single-agent case [28]), consistent with mitragynine as the proximate agent, although the pulmonary finding itself remains nonspecific.

### Exposure and Toxicology

Exposure was single-substance in 9 reports and polysubstance in 15. Mitragynine was quantified almost exclusively in forensic cases; clinical exposure was usually established by history or self-report, a notable evidence gap. Across fatal cases mitragynine concentrations ranged widely (approximately 190–7,500 ng/mL): single-drug deaths clustered at the high end [27, 28], whereas deaths with central-nervous-system-depressant co-drugs occurred at lower mitragynine concentrations. Although this pattern is compatible with additive toxicity lowering the lethal threshold, the available data cannot establish that mitragynine contributed causally to death in these polysubstance cases. Interpretation of these concentrations is further limited by post-mortem redistribution — central-to-peripheral ratios for mitragynine are variable [37] — and by analytical challenges including poor analyte stability and incomplete resolution of mitragynine from its diastereomers [38]. Concentrated or high-7-hydroxymitragynine products were implicated in two clinical cases [23, 25], aligning with the 2025 regulatory concern [4].

### Outcomes and Causality

All 14 clinical patients survived with supportive care, corticosteroids, or naloxone; all forensic decedents died. Attribution was graded Probable in 9 reports, Possible in 13, and Unlikely in 2; no report met criteria for Certain, reflecting the general absence of rechallenge. The Probable-or-better group spanned all five categories.

### Sensitivity Analyses

Restricting the synthesis to reports graded Probable or better (*n* = 9) retained at least one high-attribution exemplar in most categories, and the predominance of pulmonary edema among fatal cases persisted. An analysis restricted to single-substance exposures (*n* = 9) — a nearly identical set — likewise preserved the framework. A further analysis restricted to peer-reviewed clinical reports (excluding the 5 conference abstracts) retained two peer-reviewed reports supporting diffuse alveolar hemorrhage and one supporting ARDS, but left the eosinophilic/hypersensitivity pneumonitis category without peer-reviewed support, since both reports in that category were conference abstracts. The primary–secondary structure was retained in these sensitivity analyses, although the small sample and residual confounding preclude firm conclusions.

### Risk of Bias

On the Murad 2018 tool [7], 6 reports were judged at low concern, 12 at moderate, and 6 at high. Concern was lowest for single-drug forensic cases and for clinical cases with a thorough alternative-cause workup (including the case with a positive rechallenge), and highest where a confirmed alternative cause was present or key data (e.g., chest imaging) were missing; conference abstracts scored as unclear on reporting completeness. The full appraisal is provided in the supplementary workbook.

## Discussion

In this first synthesis of kratom-associated pulmonary toxicity, 24 reports distributed across five categories that fall into two tiers. Three primary pulmonary syndromes — diffuse alveolar hemorrhage, ARDS, and eosinophilic/hypersensitivity pneumonitis — were observed in living patients and were generally survivable with supportive care and corticosteroids. Two secondary manifestations — central respiratory depression and post-mortem pulmonary edema/congestion — reflect systemic rather than lung-directed toxicity: in respiratory depression the lung is the effector organ, and post-mortem pulmonary edema is a nonspecific terminal finding common to opioid-type deaths generally.

These observations extend the existing kratom safety literature, which has documented hepatic, cardiac, neurological, and overdose-related harms and a rising burden of exposures and deaths [3, 5], into the respiratory domain that prior organ-system reviews have largely omitted. They are concordant with forensic analyses in which kratom-positive decedents frequently show nonspecific pulmonary edema and congestion [5]; because such edema is common across drug-related deaths generally, it is best interpreted as a frequent final finding in fatal kratom toxicity rather than as evidence that the lung is a specific target organ. One included fatality illustrates the point directly: autopsy showed only mild pulmonary edema, and death was certified as aspiration of chyme after loss of consciousness attributed to combined mitragynine and etizolam toxicity [10], underscoring that the pulmonary finding may be incidental to the mechanism of death.

These categories are biologically plausible. Mu-opioid agonism by mitragynine and 7-hydroxymitragynine can produce opioid-type central respiratory depression and secondary (neurogenic/hydrostatic) pulmonary edema [1, 2], while immune-mediated mechanisms may underlie the eosinophilic and hypersensitivity presentations; increased alveolar-capillary permeability has been proposed for the hemorrhage and acute-lung-injury phenotypes. The positive rechallenge in one pneumonitis case [20] provides the strongest individual causal signal in the dataset.

The categories likely reflect distinct mechanisms acting at different doses and over different time-courses. The opioid-like phenotype is the most directly explained: mitragynine and, far more potently, 7-hydroxymitragynine are partial agonists at the mu-opioid receptor [1, 2], and at high exposures produce the same brainstem-mediated hypoventilation seen with conventional opioids, which is naloxone-reversible and can culminate in hypoxemic arrest. Sustained hypoventilation, hypoxia, and surges in sympathetic tone can in turn precipitate neurogenic and hydrostatic pulmonary edema. The eosinophilic and hypersensitivity presentations, by contrast, are more consistent with an immunologically mediated, dose-independent reaction; their corticosteroid responsiveness and the single positive rechallenge [20] favor a hypersensitivity mechanism rather than direct cytotoxicity. The alveolar-hemorrhage and acute-lung-injury phenotypes are best explained by disruption of the alveolar-capillary barrier, although whether this reflects a direct alkaloid effect, an adulterant or contaminant in unregulated products, or an idiosyncratic immune response cannot be resolved from the available reports.

That the framework was largely preserved under restriction to Probable-or-better and to single-substance exposures is reassuring, though the small numbers mean this cannot be taken to exclude an artifact of confounded reports. The single-drug fatalities [27, 28] — with high mitragynine concentrations and otherwise negative comprehensive screens — are the reports most consistent with high-concentration mitragynine exposure contributing to death in the absence of other detected drugs; the accompanying pulmonary edema, however, is a nonspecific terminal finding rather than evidence of lung-specific toxicity. The lower mitragynine concentrations seen in polysubstance deaths are compatible with, but do not establish, additive central-nervous-system depression lowering the lethal threshold.

Interpreting the forensic concentrations requires caution. Mitragynine undergoes post-mortem redistribution, and central-to-peripheral blood ratios in the included cases were variable [37], so a single concentration cannot be read as a reliable index of the ante-mortem dose. Even so, an internally consistent pattern emerged: the highest mitragynine concentrations occurred in the single-agent deaths, whereas polysubstance deaths were certified at substantially lower concentrations. This bears on the current regulatory debate. Concentrated and semi-synthetic 7-hydroxymitragynine products are markedly more potent at the mu-opioid receptor than the parent leaf alkaloid [4], and their proliferation plausibly shifts the exposure distribution toward the high-potency end implicated in the most severe presentations; two clinical cases here involved high-7-hydroxymitragynine exposure [23, 25], and the 2025 federal scheduling recommendation [4] is consistent with an opioid-type risk profile that the traditional leaf does not carry. Because the leaf and concentrated 7-hydroxymitragynine differ so markedly in potency, and because product characterization was inconsistent across reports, the two exposure types cannot be cleanly separated in this dataset — an important interpretive limitation.

These observations suggest several practical considerations. It may be reasonable for clinicians to ask specifically about kratom and 7-hydroxymitragynine products in patients with unexplained acute lung injury, alveolar hemorrhage, eosinophilic pneumonitis, or opioid-type respiratory depression, because these exposures are not detected on routine immunoassay drug screens and require targeted liquid chromatography–mass spectrometry [2]. Evaluation should still exclude infection, autoimmune disease, cardiogenic edema, aspiration, and other competing causes. Reported management was presentation-directed: naloxone for respiratory depression, lung-protective ventilation and prone positioning for acute lung injury, and corticosteroids for hemorrhage and eosinophilic phenotypes. The contrast between survivable clinical cases and fatal forensic cases is consistent with early recognition and supportive care being important, though it may also reflect selection and reporting differences between clinical and forensic sources rather than a purely biological gradient.

Several priorities follow. Future reports should adhere to standardized, structured case reporting and, wherever feasible, include quantitative toxicological confirmation of mitragynine and 7-hydroxymitragynine, since exposure in most clinical cases rested on self-report. Prospective surveillance through poison-control and forensic registries, which already capture rising kratom involvement [3, 5], could quantify the true incidence of pulmonary involvement and distinguish leaf from concentrated-7-hydroxymitragynine exposures. Mechanistic work is needed to test whether the hemorrhage and acute-lung-injury phenotypes reflect direct alkaloid toxicity, product adulteration, or idiosyncratic immune responses. As the market shifts toward high-potency products and the regulatory framework evolves, clinicians will need a shared vocabulary for these presentations; the two-tier framework proposed here is intended, provisionally, to provide one.

### Strengths and Limitations

Strengths include prospective registration, a search without language or date restriction, dual independent screening, extraction and appraisal, explicit attribution grading, and pre-specified robustness analyses.

The principal limitation is the nature of the evidence: case reports, conference abstracts, and retrospective forensic series without comparison groups. Such sources are subject to strong publication and reporting bias, being preferentially published when presentations are severe, unusual, or fatal; this synthesis therefore likely over-represents dramatic pulmonary involvement and under-represents mild or subclinical effects, and a funnel-plot assessment is not applicable to case-level narrative synthesis. Five of the 14 clinical reports (36%) were conference abstracts that had not undergone full peer review and provided limited methodological detail; in the sensitivity analysis excluding these, the eosinophilic/hypersensitivity pneumonitis category retained no peer-reviewed support. Polysubstance exposure was frequent (15 of 24 reports), and clinical exposure was usually established by self-report without analytic confirmation, so etiologic attribution to kratom is necessarily limited. The evidence also spans native-leaf and concentrated/high-7-hydroxymitragynine exposures, whose pharmacology differs substantially; with only 24 reports and inconsistent product characterization, a formal source-stratified analysis was not feasible.

Our attribution instruments have important limitations in this setting. We relied on the Naranjo scale for clinical reports and the WHO-UMC system for forensic cases; neither was developed for overdose toxicology, unregulated herbal products, or post-mortem investigation. The Naranjo scale depends heavily on rechallenge and dechallenge information rarely available in these settings, and its application to poisoned patients has been questioned [39], with poor inter-rater reproducibility in published case reports [40] and no validation for unregulated supplements [41]. The WHO-UMC system was designed for pharmacovigilance in living patients; its higher categories require dechallenge and rechallenge that are impossible at autopsy, and it does not account for post-mortem redistribution, tolerance, or the polypharmacy typical of drug-related deaths [37]. We therefore interpret these gradings as approximate ordinal descriptors of attribution strength rather than validated measures of causation, and we frame the forensic cases with reference to forensic-specific approaches to drug-death certification [42, 43]. As noted, a formal inter-rater agreement statistic was not calculated.

Two includable records were identified only by citation searching [15, 26] because their titles emphasized non-pulmonary features, suggesting that database search under-captures records indexed under multiorgan or non-respiratory terms. Finally, the categories are not mutually exclusive — central respiratory depression may coexist with, or precipitate, the pulmonary edema seen at autopsy — and the framework is intended as a pragmatic, provisional clinical aid rather than a validated mechanistic taxonomy; refining and validating it will require the larger, more completely characterized case series that standardized reporting should enable.

## Conclusions

Reported pulmonary presentations of kratom exposure clustered into five categories that fall into two tiers: primary pulmonary syndromes, in which the lung appears to be the target organ (diffuse alveolar hemorrhage, ARDS/diffuse acute lung injury, and eosinophilic/hypersensitivity pneumonitis), and secondary pulmonary consequences of systemic toxicity (central respiratory depression and post-mortem pulmonary edema/congestion). Because the evidence derives almost entirely from case reports, conference abstracts, and retrospective forensic series, these categories should be regarded as provisional and hypothesis-generating rather than established phenotypes. Nonetheless, they may offer clinicians a practical vocabulary for recognizing kratom- and 7-hydroxymitragynine-associated respiratory presentations. Routine elicitation of kratom use, targeted toxicological confirmation, and standardized reporting of future cases are needed to test and refine this framework and to define the true burden of pulmonary involvement.

## Data Availability

The full data-extraction, risk-of-bias, and synthesis workbook is available from the corresponding author and is provided as a supplementary file.
